# Advantages and Disadvantages of Aluminum as Applied to Dentistry

**Published:** 1868-09

**Authors:** George O. Starr

**Affiliations:** New York


### ADVANTAGES AND DISADVANTAGES OF ALUMINUM AS APPLIED TO DENTISTRY.

BY GEORGE O. STARR, OF NEW YORK.

Since aluminum has been made useful to the Dental profession by the discovery of Dr. A. Starr, of this city, of a solder for that metal, it is desirable for all Dentists to be fully satisfied of its good qualities, which to some are entirely unknown. I give the following as my observations, which have been made during the last two years, in regard to its advantages and disadvantages as compared with other bases for artificial teeth. As compared with gold, which many consider as the best article upon which to insert artificial teeth: the advantages which gold possesses over aluminum is greater strength and capabilities of a higher finish; while aluminum has the advantage over gold in being only about one-sixth its specific gravity; is more easily worked, and is capable of resisting the action of the alkalies and acids of the mouth nearly as well as gold. What has been said of gold as compared with aluminum, will apply to platinum as well. Aluminum in comparison with silver has many advantages. Silver will corrode, while aluminum will not. Silver has four times the specific gravity, and has about the same strength, but is not as easily adapted to the mouth as aluminum. Rubber is not to be compared with aluminum; it is thicker, heavier, more brittle, no cheaper, and lastly, unhealthy as a hundred physicians will testify. Besides all this, there are many, so-called Dentists, undeserving of the name, who can do no other kind of work, and who by their use of the compound called rubber have done immense injury to the profession.

If scientific and educated Dentists would refuse to countenance the use of rubber, and use that article which has all and more good qualities than is claimed for rubber; I mean, aluminum which requires a thorough Dentist to work it as it should be, it would result in much benefit, both to themselves and the public, and the complete downfall of inexperienced men who claim to be Dentists, and the defeat of the rubber company.

